# Supplementary material for: ChatGPT as a Tool for Biostatisticians: A Tutorial on Applications, Opportunities, and Limitations
Source: Stat Med. 2025 Oct 23;44(23-24):e70263. doi: 10.1002/sim.70263 (PMC12548020; doi:10.1002/sim.70263)
Supplement: Supplementary file 1 — Data S1. Supporting Information. [file SIM-44-0-s001.zip › Supplementary Materials Additional.zip!/3.4_Sample_Sizes_Planning_for_Survival_Outcomes/3.4_Sample_Sizes_Newer_Versions/03_chat_sample_size_o4mini.pdf]

03_chat_sample_size_o4mini

Please clear memory

I don't have the ability to manually wipe my internal memory, but each session's context resets once our conversation ends. If you'd like to start fresh right now, we can simply shift the topic or I can disregard prior messages—just let me know how you'd like to proceed!

You take the role of an expert biostatistician in this chat.

Understood. I'll adopt the role of an expert biostatistician. How can I assist you today—data analysis, study design, statistical modeling, or something else?

I would like to compare a new therapy with the standard therapy regarding survival in advanced pancreatic carcinoma. Please perform a sample size calculation and incorporate the following parameters:
o Primary outcome: Overall survival.
o Median survival time in the standard therapy group: 12 months.
o Median survival time in the new therapy group: 18 months.
o Power = 80% (1 - β = 0.8)
o a two-sided significance level of α = 0.05.
o The allocation ratio should be 1:1 (equal group sizes).
o Patients will be followed up for 2 years.
o We expect a dropout rate of 15%.
Use the data analysis mode

Assuming exponential survival and uniform accrual, here's how the two-arm log-rank sample size works out:

1. **Inputs & hazard ratio**
    - Median OS (standard): 12 months ⇒ hazard rate λ_1 = ln 2 / 12
    - Median OS (new): 18 months ⇒ hazard rate λ_2 = ln 2 / 18
    - ⇒ hazard ratio HR = λ_2/λ_1 = (ln 2/18)/(ln 2/12) = 12/18 ≈ 0.667
    - Two-sided α=0.05 ⇒ $Z_{1-}\alpha/_2$ = 1.96
    - Power 80% ⇒ $Z_{1-}\beta$ = 0.84
2. **Required number of events (Freedman's formula)**

$$E = \frac{(Z_{1-\alpha/2} + Z_{1-\beta})^2}{(\ln HR)^2} = \frac{(1.96 + 0.84)^2}{[\ln(0.667)]^2} \approx 47.7$$

3. **Expected event proportion over 2 years**
   - Standard arm:
     $p_1 = 1 - e^{-\lambda_1 \cdot 24} = 1 - e^{-(\ln 2/12) \cdot 24} = 0.75$
   - New arm:
     $p_2 = 1 - e^{-\lambda_2 \cdot 24} = 1 - e^{-(\ln 2/18) \cdot 24} \approx 0.603$
   - Average event rate = (0.75 + 0.603)/2 ≈ 0.677
4. **Total sample size (no dropout)**

$$N = \frac{E}{\text{avg event rate}} = \frac{47.7}{0.677} \approx 70.6$$

   ⇒ round up to 71 subjects total (≈ 36 per arm)

5. **Inflate for 15% dropout**

$$N_{\text{adj}} = \frac{N}{1 - 0.15} \approx \frac{70.6}{0.85} \approx 83.0$$

   ⇒ round up to **84 subjects total**, i.e. **42 per arm**.

Conclusion:
You will need to enroll **84 patients** (42 in each arm) to detect an increase in median OS from 12 to 18 months with 80% power at a two-sided 5% level, allowing for 2 years of follow-up and a 15% dropout.

please check whether the formular for the required number of events is correct. Did you incorporate the proportions of group size?

You're right to suspect that the simple Freedman formula I used

$$E = \frac{(Z_{1-\alpha/2} + Z_{1-\beta})^2}{(\ln HR)^2}$$

applies only in the special case of **equal allocation** when one implicitly sets $p_1 = p_2 = ½$ and then absorbs the factor $p_1 p_2 = 0.25$ into the numerator. In the general two-arm setting with allocation fractions p_1 and $p_2 = 1 - p_1$, the correct formula for the **total number of events** required is

$$E = \frac{(Z_{1-\alpha/2} + Z_{1-\beta})^2}{p_1 \, p_2 \, (\ln HR)^2}.$$

---

1. Allocation proportions

- Here $p_1 = p_2 = 0.5$, so

$$p_1 p_2 = 0.5 \times 0.5 = 0.25.$$

2. Re-compute required events

- $HR = \dfrac{\ln(2)/18}{\ln(2)/12} = 12/18 = 0.667.$
- $Z_{1-\alpha/2} = 1.96$, $Z_{1-\beta} = 0.84.$
- $\ln HR = \ln(0.667) \approx -0.4055.$

Plugging in:

$$E = \frac{(1.96+0.84)^2}{0.25\,(-0.4055)^2} = \frac{2.80^2}{0.25 \times 0.1644} = \frac{7.84}{0.0411} \approx 191 \text{ events.}$$

---

3. Translating events to sample size

As before, over 24 months of follow-up we expect:

- **Standard arm event rate**
  $p_1 = 1 - e^{-\lambda_1 \cdot 24} = 1 - e^{-(\ln 2/12)\cdot 24} = 0.75.$
- **New arm event rate**
  $p_2 = 1 - e^{-\lambda_2 \cdot 24} = 1 - e^{-(\ln 2/18)\cdot 24} \approx 0.603.$
- **Average**
  $\bar{p} = (p_1 + p_2)/2 \approx 0.6765.$

Thus the **total number of patients** needed (ignoring drop-out) is

$$N = \frac{E}{\bar{p}} = \frac{191}{0.6765} \approx 282 \text{ patients.}$$

With a **15%** anticipated dropout:

$$N_{\text{adj}} = \frac{282}{1-0.15} \approx 332 \text{ patients total}$$

i.e.\ **166 per arm**.

---

4. Summary

- The original Freedman formula must include the factor $p_1 p_2$.
- With equal (1:1) allocation, $p_1 p_2 = 0.25$, yielding **≈191 required events**, not 48.
- Translating to patients (2 year follow-up, 15% dropout) gives **332 total** (166 per arm).

Let me know if you'd like to explore alternative accrual or drop-out assumptions, or different survival models!
